# Viewing oxidative stress through the lens of oxidative signalling rather than damage

**DOI:** 10.1042/BCJ20160814

**Published:** 2017-03-07

**Authors:** Christine H. Foyer, Alexander V. Ruban, Graham Noctor

**Affiliations:** 1 Centre for Plant Sciences, School of Biology, Faculty of Biological Sciences, University of Leeds, Leeds LS2 9JT, U.K.; 2 School of Biological and Chemical Sciences, Queen Mary University of London, Mile End Road, London E1 4NS, U.K.; 3 Institute of Plant Sciences Paris-Saclay IPS2, Univ Paris Sud, CNRS, INRA, Univ Evry, Univ Paris-Diderot, Sorbonne Paris-Cite, Universite Paris-Saclay, Rue de Noetzlin, Orsay 91405, France

**Keywords:** cell signalling, oxidative stress, photoinhibition, photosynthesis, reactive oxygen species

## Abstract

Concepts of the roles of reactive oxygen species (ROS) in plants and animals have shifted in recent years from focusing on oxidative damage effects to the current view of ROS as universal signalling metabolites. Rather than having two opposing activities, i.e. damage and signalling, the emerging concept is that all types of oxidative modification/damage are involved in signalling, not least in the induction of repair processes. Examining the multifaceted roles of ROS as crucial cellular signals, we highlight as an example the loss of photosystem II function called photoinhibition, where photoprotection has classically been conflated with oxidative damage.

## Introduction

Redox signalling is an essential component of cellular energy homeostasis and responses to the environment in animals and plants, redox-sensitive proteins functioning as sensors that trigger repair mechanisms and regulate cell division, growth and defence processes. Despite a growing acceptance in the animal and plant literature that ROS accumulation and programmed cell death are not the enemy but rather hallmarks of survival [1,2], old paradigms die hard. The concept that ROS mediate their principal effects by causing indiscriminate irreversible inactivation of proteins and/or loss of function of other cellular components (i.e. damage) became strongly anchored within the literature with the advent of initiatives to confer general stress tolerance on plants by overexpression of antioxidative enzymes. This notion remains surprisingly persistent to this day, probably because it is extremely simple. According to the ‘damage’ paradigm, overproduction of ROS in conditions such as excess light availability induces a general loss of cellular functions through processes such as photoinhibition, lipid peroxidation and protein oxidation, the accumulation of damage leading ultimately to death. Not before time, this simple paradigm is finally being laid to rest. Evidence that is often cited in apparent support of the ‘reduction good/oxidation bad’ paradigm is that oxidation leads to loss of enzyme activity. However, such effects have often only been demonstrated *in vitro*, sometimes using oxidant concentrations that are not biologically relevant. Furthermore, the literature contains physiologically relevant counterexamples, such as oxidative activation of chloroplast glucose-6-phosphate dehydrogenase [3,4] and protein kinase signalling cascades [5]. Crucially, the biological relevance of oxidative changes must be understood within the context of cellular functions: loss of activity of a given protein may activate a function at the cellular level.

## ROS at the heart of intracellular and cell-to-cell signalling

ROS play numerous important roles in plant development and environmental responses. ROS functions in plants are tightly intertwined with signalling pathways through phytohormones. It has long been apparent from studies of plant responses to the gaseous pollutant ozone that ROS interact with stress hormones such as ethylene, salicylic acid and jasmonic acid [6]. More recently, it has been established that ROS and related molecules such as thiols interact with auxins, gibberellins and cytokinins to control plant growth and development [7–9]. ROS-related redox processes probably intervene at multiple levels of signalling for any given phytohormone. For example, thiol-dependent steps are probably involved in both synthesis and signalling of salicylic acid [10]. In this regard, ROS can act either as a life or a death signal, dependent on the molecular and cellular context in which ROS accumulate. The outcomes of such signalling depend on many parameters, principally the chemical nature of the ROS form produced (i.e. superoxide, hydrogen peroxide or singlet oxygen) and the nature of the interacting partner (protein thiol, metabolite, lipid or DNA molecule), as well as cell identity, but all types of oxidative modification (reversible or irreversible) can be viewed as part of the redox signalling matrix because they induce regulatory, repair or death responses. Together with cellular oxygen tension, ROS control many crucial aspects of animal and plant biology, not least cell proliferation, stem cell homeostasis and differentiation lineage commitment [11–14].

Publications continue to appear that are based on the premise that low doses of ROS play beneficial roles in signalling, while higher doses have detrimental effects. Cell death is often cited as an example of the latter, supposedly undesirable effects, even though this process is central to renewal, immunity and defence responses. In multicellular organisms, the elimination of certain cells is a beneficial process, whether it is in control of uncontained cell division in mammalian cells or in the hypersensitive response in the case of plants resisting pathogen attack. Even at the local (cellular) level, there is abundant evidence that cell death in plants does not only occur generally through damage that overwhelms the cell's defences, but rather through genetically programmed pathways, with signalled processes that are controlled by specific genes and that may involve the programmed withdrawal of antioxidative systems. The importance of specific ‘executor’ genes was first reported in plants accumulating singlet oxygen [15]. Genetic control over ROS-induced cell death is equally apparent from studies of the effects of H_2_O_2_ in catalase-deficient plants, using both reverse and forward genetics [16–18].

## ROS and photosynthesis

**Figure 1.**
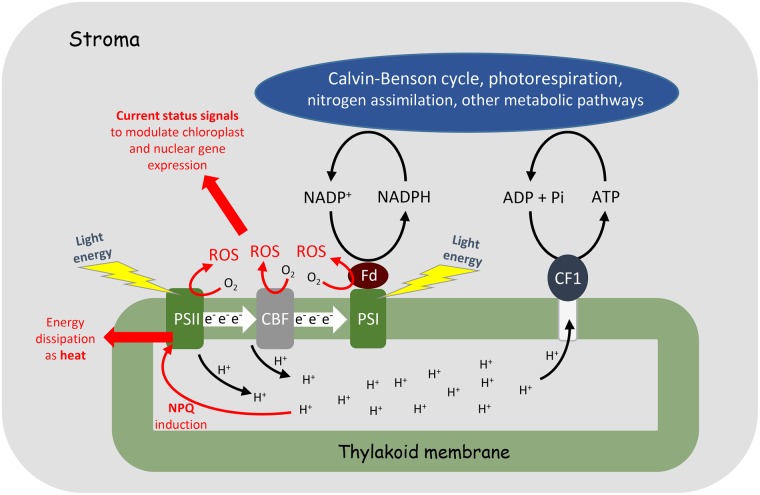
Matching supply and demand in photosynthesis. Light energy drives otherwise thermodynamically unfavourable electron transfer at PSI and PSII to enable the reduction in ferredoxin (*F*_d_) and NADP^+^ in the stroma. Electron transfer is accompanied by the release of protons into the intrathylakoid space during water-splitting at PSII and plastoquinol oxidation at the cytochrome b_6_f complex (CBF). The protons are used by the coupling factor to produce ATP which, together with the NADPH generated from electron transport, drives metabolism in the stroma. If the proton concentration inside the thylakoid reaches a certain value, non-photochemical quenching (NPQ) mechanisms are activated to enable energy dissipation as heat. Oxygen oils the wheels of the whole process: the continuous production of ROS at various sites in the electron transport chain serves numerous functions, including contributing to the proton gradient required for ATP generation, redox poising (adjustments of the ratios of reduced to oxidised forms of electron transfer components) and providing information on current status through signalling pathways.

Chloroplasts were one of the very first sources of superoxide and H_2_O_2_ to be described in plants [19,20]. Electron flow to oxygen rather than NADP^+^ relieves reductive pressure within the electron transport chain and balances ATP:NADPH ratios by allowing proton pumping without net reductant generation (Figure 1). Moreover, like ROS formed at other subcellular locations, thylakoid-generated ROS may play crucial roles as signal transducers (Figure 1). ROS generated during light capture and electron transport are situated at the interface between the environment and the molecular machinery of photosynthesis, thereby providing the cell with crucial information on current status [21,22].

As well as superoxide and H_2_O_2_ production by the photosynthetic electron transport chain, energy transfer within the photosystems leads to generation of singlet oxygen, a ROS that is formed by excitation energy transfer from triplet chlorophyll to O_2_ [23,24]. The high reactivity of membrane proteins and lipids to singlet oxygen makes these prime targets for signalling from photosystem II (PSII) to the nucleus. While oxidative modification of PSII structural and repair proteins and lipids is unavoidable [25,26], considerable uncertainty remains concerning the extent to which such processes impair PSII function within a physiological context.

## Photoinhibition and regulation of PSII

The management of light interception and energy conversion is one of the most fundamental concepts in understanding the regulation of photosynthesis. The chloroplast is faced with the problem of balancing light harvesting with the generation of ATP and NADPH, in appropriate ratios, at rates that match the demands of metabolism [27]. The fast turnover of these pools in the light leaves little room for imbalances in rates of energy production and consumption, explaining the evolution of a plethora of stabilising mechanisms that come into play, as required, to ensure smooth running of the system over the wide range of irradiances that occur in the natural and field environments. Such mechanisms include pH-triggered non-photochemical chlorophyll fluorescence quenching (NPQ) at PSII and the direct transfer of energy and electrons to oxygen leading to the production of reactive oxygen species (ROS) such as singlet oxygen, superoxide and H_2_O_2_ (Figure 1), all of which play important roles in the regulation of photosynthesis [27].

An extensive body of literature has accumulated over the last 30 years on phenomena that are included under the term ‘photoinhibition’. Considerable confusion persists because this term includes both photodamage and down-regulation of PSII function. Even though increasing evidence suggests that photodamage is not generally the dominant component, photoinhibition tends still to be equated with ‘damage’. For this reason, in the following critique, photoinhibition is used to denote this long-standing notion of ‘photodamage’.

Excess sunlight saturates PSII, causing build-up of excess excitation energy in its antenna. This unused energy is potentially dangerous because it can lead to the inactivation of the reaction centres (RCIIs), resulting in a sustained decrease in the quantum efficiency of PSII and the subsequent electron transport rate, a phenomenon termed photoinhibition [28–30]. Indeed, the photosynthetic pigments of oxygen-evolving PSII should be potentially vulnerable to photoinhibition since the RCII possesses a very strong oxidative potential of ∼1.17 V that is required to oxidise water. Under conditions where electron donation to P680 is less efficient than its photo-oxidation, an increase in the P680^+^ lifetime will occur. This powerful oxidant may oxidise the nearest pigments and amino acids, causing their degradation and a subsequent degradation of the key RCII D1 protein [29]. In other circumstances, when the acceptor side is less efficient, a radical pair will be formed. The recombination of this pair will lead to the formation of a P680 triplet state that was proposed to interact with atmospheric triplet oxygen, causing formation of highly reactive singlet oxygen, which in turn can lead to the degradation of the key RCII component, D1 protein [30–33]. Hence, initially photoinhibition was thought to lead to a decreased number of active RCIIs.

Mechanisms to deal with high light exposure are required to minimise the build-up of potentially photodamaging excess energy in PSII. An imbalance between ATP generation and utilisation, caused, for example, by a failure of metabolism to keep pace with the thylakoid reactions, will cause protons to rapidly accumulate in the intrathylakoid space (Figure 1). This leads to decreased PSII light-harvesting efficiency through a process called NPQ that protects RCII from the damage via prompt dissipation of excess energy as heat [34]. Apart from being triggered by the proton gradient (ΔpH), NPQ is strongly enhanced by the xanthophyll cycle activity that leads to conversion of violaxanthin into zeaxanthin. This reaction is also dependent on the ΔpH, albeit on a somewhat slower timescale than onset of either ΔpH or, in many cases, the pH-dependent NPQ. Importantly, PSII quantum efficiency can be decreased by both photodamage to RCII and NPQ (see below). Until recently, the only criterion that was used to separate photodamage from the protective reduction in the PSII yield (otherwise called *down-regulation*) was the timescale of the recovery of these processes in the dark. Indeed, while the repair from the damage to RCIIs takes hours, the down-regulation via NPQ was believed to take only minutes to recover [33]. The evidence for the former was taken from biochemical data (D1 protein repair) [34], whereas the evidence for the latter was taken from the so-called pulse amplitude-modulated chlorophyll fluorescence analysis, the interpretation of which remains a source of controversy [35].

## Interpreting chlorophyll fluorescence

**Figure 2.**
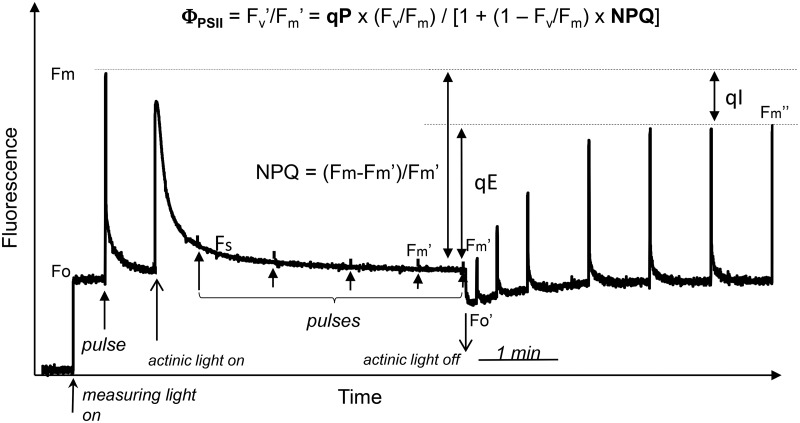
A typical pulse amplitude-modulated chlorophyll fluorescence induction measurement. The modulated low intensity measuring light of ∼1 µmol m^−2^ s^−1^ is used to excite chlorophylls of the PSII antenna fluorescence (*F*_o_ level). In these conditions, fluorescence is highly quenched by working RCs (RCIIs). Application of a saturating light (10 000 µmol m^−2^ s^−1^) for 1 s causes the closure of all reaction centres for the measuring light so that they stop quenching of the antenna fluorescence and the level rises to *F*_m_. After ∼1 min, a continuous illumination is applied (actinic light) of an intensity of ∼800 µmol m^−2^ s^−1^. This causes gradual quenching of *F*_m_ to the *F*′_m_ level. This quenching is triggered by the proton gradient and called NPQ. After ∼5 min, the actinic light is turned off and NPQ begins to recover. The recovery that is not complete within 5 min of darkness is termed *qI*.

Chlorophyll fluorescence has been used for several decades for prompt and non-destructive assessment of PSII efficiency in a variety of photosynthetic organisms [34,36]. A typical fluorescence-quenching experiment is depicted in Figure 2. The active (open) PSII RCIIs are efficient quenchers of antenna chlorophyll fluorescence (*F*_o_) excited by a very weak light (*measuring light*). Application of a saturating light pulse (10 000 µmol m^−2^ s^−1^) for 1 s closes all RCIIs, bringing the fluorescence to the *F*_m_ level. Now, the quantum efficiency of PSII can be expressed as Φ_PSII_ = (*F*_v_)/*F*_m_, where *F*_v_ = *F*_m_ − *F*_o_. Application of continuous illumination for 5 min causes a gradual decline not only in *F*_s_, a steady-state fluorescence level, but also in *F*_m_, which becomes *F*′_m_. The decline in *F*_m_ is called non-photochemical quenching (NPQ) and is often expressed as NPQ = (*F*_m_ − *F*′_m_)/*F*′_m_. NPQ decreases the yield of PSII under continuous illumination that can now be expressed as

1 $$\Phi_{\mathrm{PSII}}=\frac{F_{v}^{\prime }}{F_{m}^{\prime }}=qP\times \frac{\left(F_{v}/F_{m}\right)}{\left[1+(1-F_{v}/F_{m})\times \mathrm{NPQ}\right]},$$

where *qP* is photochemical quenching [*qP* = (*F*′_m_ − *F*_s_)/(*F*′_m_ − *F*′_o_)]. Hence, the yield of PSII is a function of both NPQ and *qP*. In the dark, following moderate levels of illumination, *qP* = 1 and NPQ recovers gradually but not completely (Figure 2). The slowly reversible NPQ component is called *qI* [equal to (*F*_m_ − *F*_m_″)/*F*_m_″] and was first proposed to reflect the photodamage to RCII [36,37] diminishing the PSII yield in the dark as can be seen from the formula (1). However, later it was discovered, mainly by the groups of Adams and Demming-Adams, that a large part of *qI* does not reflect the damage to RCIIs but relates to the synthesis of zeaxanthin, and promotion of the slowly reversible NPQ components [38,39] at high light and low temperature conditions [40–43]. Hence, the PSII yield in the classical fluorescence measurements was established to be dependent on both the RCII photodamage and the sustained NPQ. To quantify the true effect of photodamage upon Φ_PSII,_ a new method has been developed [44,45]. It uses a gradually increasing actinic light illumination and the periodic measurements of Φ_PSII_ [(*F*′_m_ − *F*_s_)/*F*′_m_] and compares them with the analytically derived Φ_PSII_ using the formula (1). The measured and calculated values of Φ_PSII_ match each other very well at somewhat low actinic light intensities, but gradually fall apart at somewhat higher light. This disparity can be easily corrected if the *qP* values in the dark were <1. Indeed, these values have been obtained using the comparison between measured and calculated values of *F*′_o_ [46]. Therefore, the *qP* in the dark (*qP*_d_) has been proposed as a true indicator of photoinhibition reflecting the percentage of the inactivated RCIIs [46]. The use of this parameter demonstrated that a prolonged exposure to the saturating light intensities (1500–2000 µmol m^−2^ s^−1^) only caused inhibition to at most 25% of PSII RCIIs of established mature plants [45,47]. Hence, photoinhibition may not be a common phenomenon in nature. If the D1 repair process is ongoing and recovery without damage is facilitated by the protective component of NPQ, the loss of the PSII yield due to photoinhibition will greatly decrease [45,47]. Therefore, the sustained decline in *F*_v_/*F*_m_ should be interpreted as predominantly related to PSII down-regulation via sustained components of protective NPQ and not as damage resulting from photoinhibition, and the *qP*_d_ parameter should be used to quantify this change [44,45,47,48].

## Conclusions and perspectives

The Manichean notion that sets evil ROS on one side and benevolent antioxidants on the other is impossible to defend. Different oxidants may antagonise each other, and antioxidants such as glutathione may play an integral part not only in controlling ROS but also in transmitting oxidative signals [10,49]. Enzymes that play important antioxidative roles can also promote ROS production or ROS-dependent processes [50,51]. While plant cell functions operate at rather negative redox potentials in the soluble phase [21,22], the paradigm that oxidation is bad, while reduction is good, is too simplistic given the complexity of redox interactions [52]. This is particularly true in photosynthesis, which is driven by large redox and energy gradients, and which is the major source of ROS in plant cells. ROS production, signalling and removal associated with photosynthesis provide flexibility and control in the management of high light stress. Grasping the implications of this paradigm shift is key to addressing global issues such as food security and the production of crops in a sustainable manner for a growing world population. This challenge has led to an upsurge of interest in improving photosynthesis through the manipulation of processes that alter the light use efficiency of photosynthesis [48]. Current initiatives such as the introduction of characteristics of C_4_ photosynthesis into important C_3_ species such as rice and, more generally, improving the capture of light energy and its conversion into biomass [45,53] might benefit from an enlightened appreciation of the beneficial roles of ROS as a central integrator of functions at the cellular and whole-plant level.

## Abbreviations

PSII: photosystem II
RCIIs: reaction centres
ROS: reactive oxygen species.
